# Association between *CLOCK* Gene Polymorphisms and Insomnia Risk According to Food Groups: A KoGES Longitudinal Study

**DOI:** 10.3390/nu15102300

**Published:** 2023-05-13

**Authors:** Sunghee Lee

**Affiliations:** Department of Food and Nutrition, College of Health Science, Kangwon National University, Samcheok 25949, Republic of Korea; sunglee@kangwon.ac.kr

**Keywords:** insomnia, food groups, *CLOCK* gene

## Abstract

Food intake could mitigate or exacerbate the risk for insomnia associated with the *CLOCK* gene. This study investigated the associations between the clock circadian regulator (*CLOCK)* polymorphisms rs12649507 and rs4580704 and the risk of insomnia, as well as its interactions with food groups. Among 1430 adults, new insomnia cases were identified between 2005 and 2012. Single nucleotide polymorphisms were genotyped, and dietary intake was assessed. Next, Cox proportional hazard models were established. The fruit and meat groups significantly mitigated the risk of insomnia associated with rs12649507 among males (*p*_interaction_ = 0.006 in a recessive model; *p* = 0.010 in a dominant model). In contrast, among females the beverage group significantly increased the risk of insomnia (*p* = 0.041 in a dominant model). As for rs4580704, among males the fruit and meat groups modified the risk of insomnia (*p* = 0.006 in a recessive model; *p* = 0.001 in a dominant model). However, among females, the beverage group exacerbated the risk of insomnia associated with rs4580704 (*p* = 0.004 in a dominant model). In this longitudinal study, we observed a significantly modified insomnia risk associated with the *CLOCK* gene depending on food groups. Notably, in a general population the risks were modified according to both the fruit and meat intake among 775 males but exacerbated with beverage intake among 655 females.

## 1. Introduction

Insomnia, one of the most prevalent sleep-related disorders, has increased in recent years [1,2]. A study showed that the prevalence of insomnia was 22.1% among 10,094 U.S. adults in 2008−2009 [3]. Another study reported that the prevalence of insomnia was approximately 10−20% [4], and a longitudinal study reported an incidence of 13.9% among adults [5]. Moreover, cases of insomnia have been more frequent in females [5]. Individuals with insomnia often report having difficulties in initiating sleep, waking frequently during the night, or waking up too early in the morning [6,7]. Insomnia affects not only psychological health, but also physical health conditions: it increases the risk of hypertension [8] and cardiovascular events [9]; decreases cognitive performance [10,11]; increases the likelihood of depression [12]; and contributes to overall mortality [13].

The etiology of insomnia has also been attributed to genetic factors. Specifically, the clock circadian regulator (*CLOCK*) gene, also known as the circadian locomotor output cycles protein kaput, plays an important role in regulating circadian rhythms [14]. The *CLOCK* gene was significantly associated with sleep disorders and poor sleep quality due to circadian disruption [14,15,16,17]. Specifically, the *CLOCK* rs12649507 polymorphism was associated with sleep duration [14], and individuals with the G allele indicated a poorer quality of sleep [15]. Moreover, another study showed that climacteric females with *CLOCK* rs12649507 homozygous for *TT* had a 1.78 times higher likelihood of insomnia (95% confidence interval [CI]: 1.16−2.75) [16]. A review of *CLOCK* polymorphisms from 16 studies reported that rs12649507 was linked to short sleep duration or poor sleep quality, and that rs4580704 was associated with increased energy intake due to the misalignment of circadian rhythms [17]. Another study demonstrated that individuals carrying the *CLOCK* rs4580704 minor C allele had reduced energy intake [18]. Furthermore, *CLOCK* rs4580704 was associated with metabolic syndrome [18], diabetes [19], obesity [20], and cardiovascular diseases [19].

In addition, dietary factors were reported to be associated with insomnia. Individuals who followed the Dietary Approach to Stop Hypertension (DASH) diet had a reduced likelihood of insomnia (odds ratio (OR) = 0.51, 95% CI: 0.26−1.00) [21]. Moreover, a Mediterranean diet was linked to a reduced likelihood of having insomnia [22,23]. Furthermore, insomnia symptoms were associated with inadequate nutritional intake [24].

However, despite the uncovered link between food intake and the risks of insomnia associated with the *CLOCK* gene, no study has examined the impact of food groups on the association between the *CLOCK* gene and insomnia risks in the general population. Therefore, this prospective cohort study aimed to investigate whether the consumption of particular food groups significantly modified the insomnia risk associated with the *CLOCK* gene rs12649507 and rs4580704 polymorphisms, using the data of 1430 participants (775 males 655 females aged 43−73 years) from the Korean Genome Epidemiology Study (KoGES), a longitudinal study performed over 6 years.

## 2. Materials and Methods

### 2.1. Study Population

**Figure 1:**
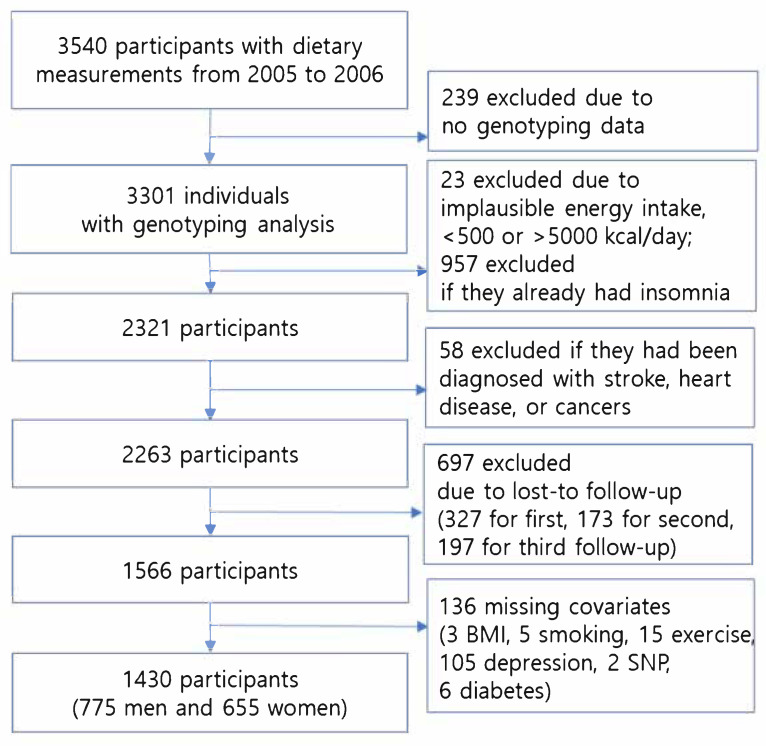
Flowchart of the study participants.

KoGES, an ongoing longitudinal prospective cohort study, was initiated to investigate the risks for chronic diseases in the general population of South Korea; at baseline, participants in the Ansan district were randomly enrolled through in-person visits, postal correspondence, and telephone conversations in 2001−2002. They were asked to visit every other year to complete questionnaires and undergo a physical examination. They also underwent venipuncture early in the morning after fasting for more than eight hours, and their blood samples were analyzed (Seoul Clinical Laboratory, Republic of Korea). All participants provided their informed consent. This study abided by the tenets of the Declaration of Helsinki. The Human Subjects Review Committee and the Institutional Review Board of Kangwon National University approved this study (KWNUIRB-2022-06-007). The data for this study was obtained from the Korea National Biobank of the Korea Disease Control and Prevention Agency. The data was publicly available and open to scientists (NBK-2022-076). To investigate which food groups might modify the insomnia risks associated with *CLOCK* polymorphisms in the KoGES cohort, 3540 participants who had undergone genotyping analysis completed a dietary analysis from 1 March 2005, to 17 December 2006. Of these participants, 239 individuals were excluded because of a lack of genotyping data. Among the 3301 individuals with both dietary and genotyping data, 23 were excluded due to implausible ranges of daily energy intake (<500 kcal or >5000 kcal). Additionally, 957 adults who already had insomnia were excluded, as the risk of insomnia was being investigated. Of the remaining 2321 individuals, an additional 58 were excluded due to a diagnosis of myocardial infarction (*n* = 13), coronary artery disease (*n* = 12), dementia (*n* = 1), stroke (*n* = 9), or cancer (*n* = 23), respectively. Overall, of the 3540 participants at baseline, 3255 (91.9%) in 2007/2008, 3262 (92.1%) in 2009/2010, and 3052 (86.2%) in 2011/2012 completed their follow-up. From 2005/2006 to 2011/2012, 697 participants were lost to follow-up and 136 were missing data (body mass index (*n* = 3), smoking (*n* = 5), exercise (*n* = 15), depression (*n* = 105), single nucleotide polymorphism (SNP, *n* = 2), and diabetes (*n* = 6)). Therefore, a total of 1430 individuals (775 males and 655 females) were included in the analyses (Figure 1).

### 2.2. Definition of Insomnia

Insomnia cases were identified based on the four symptomatic domains identified in the fourth edition of the Diagnostic and Statistical Manual of Mental Disorders (DSM-IV) published by the American Psychiatric Association [3,4,6,7]. The four domains were as follows: non-restorative sleep, difficulty initiating sleep, difficulty maintaining sleep, and waking early in the morning [6,7]. Insomnia was defined as at least one of these symptoms occurring three times per week [6,7].

### 2.3. Dietary Measurement

To examine dietary intake, study participants completed a semi-quantitative food frequency questionnaire (FFQ) that was developed by the Korea Centers for Disease Control and Prevention [25]. The questionnaire included 106 food items for which participants indicated the frequency of servings: never; once a month; 2–3 times per month; 1–2 times, 3–4 times, or 5–6 times per week; once a day; twice a day; and ≥3 times per day. The validity and reproducibility of the FFQ has been previously examined [25]. Both nutrient and energy intake were calculated based on the database of the Rural Development Administration of Korea [26]. The residual method was adopted to adjust total energy intake [27]. Based on the included ingredients and nutrients, eight food groups were categorized [28]: refined grains (e.g., rice, ramen, noodles, dumplings, rice cakes, bread, jam, cakes, and cookies; mixed grains (e.g., whole grain rice with multi-grains); vegetables (e.g., cabbage, spinach, seasoned bracken, radishes, cucumbers, lettuce, and tomatoes); fruits (e.g., strawberries, apples, grapes, melons, peaches, pears, bananas, kiwis, and oranges); meats (e.g., pork, beef, chicken, and eggs); seafood (e.g., mackerel, grilled yellow croaker, anchovies, squid, and shrimp); dairy (e.g., milk, yogurt, ice cream, and cheese); and beverages (e.g., soy milk, coffee, soft drinks, green tea, and others) (Supplemental Table S1).

### 2.4. Genotyping Analysis

The detailed processes of the genotyping analysis have been previously reported [29]. Fasting blood samples of the participants were genotyped with 50 ng of Genome-Wide Human Affymetrix 5.0 (Santa Clara, CA, USA). For quality control, missing gene call rates > 5%, minor allele frequencies (MAF) < 0.05, and *p* values of Hardy–Weinberg equilibrium (HWE) < 10^−6^ were considered [29]. The call rates of DNA genotyping were >95%. Two candidate SNPs, rs12649507 and rs4580704, of *CLOCK* polymorphisms that identified a genetic susceptibility of *CLOCK* for sleep disorders were chosen based on previous findings [14,18,30]. We identified and determined SNPs and analyzed additive, dominant, and recessive models of *CLOCK* rs12649507 and rs4580704 [14,18,30].

### 2.5. Statistical Analysis

Incident events and person-years were assessed from baseline to the end of the study follow-up period, the onset of insomnia, or death, whichever occurred first. The analyses were stratified by sex (*p*_interaction_ = 0.025). The assumption of hazard proportionality was tested and confirmed. To estimate the insomnia risk associated with *CLOCK* polymorphisms modified by each food group during follow-up, Cox proportional hazard models were established to estimate hazard ratios (HRs) with 95% CIs, after adjusting for age, smoking status, alcohol consumption (current, ex/non-drinker), body mass index (BMI), total caloric intake (kcal), depression (Beck’s Depression Index, BDI), physical activity (metabolic equivalent tasks a week, MET), C-reactive protein level (C-RP, mg/L), hypertension, and diabetes. A *p*-value of <0.05 was considered statistically significant. Analyses were conducted using PLINK v1.09 [31] and SAS 9.4 (SAS Inc., Cary, NC, USA).

## 3. Results

The characteristics of the 1430 participants (775 males and 655 females) are listed in Table 1. The participants who developed insomnia were older than those who did not (53.11 vs. 51.65 years, *p* < 0.001). Individuals with insomnia were less likely to consume alcohol than individuals without insomnia (47.62% vs. 53.79%, *p* = 0.031); additionally, those who developed insomnia scored higher on the depressive index than those who did not (*p* < 0.001). Females with insomnia had a higher prevalence of diabetes than females without insomnia (15.93% vs. 10.49%, *p* = 0.044).

The genetic characteristics of the two polymorphisms, rs12649507 and rs4580704, are listed in Table 2. The MAFs of rs12649507 and rs4580704 were 0.3598 and 0.2589, respectively. The *p*-values of HWE were 0.3556 for rs12649507 and 0.5794 for rs4580704.

To assess the risk of insomnia associated with the *CLOCK* polymorphisms, additive, dominant, and recessive models were established. Table 3 presents the risk of developing insomnia over 6 years of follow-up in each model. In the rs12649507 recessive model, males with the GG genotype had a significantly higher risk of developing insomnia than males with the A allele (HR = 1.50, 95% CI: 1.11–2.02). In the rs4580704 additive and recessive models of rs4580704, males with the GG genotype had significantly higher risks of insomnia than males with the C allele (HR = 1.51, 95% CI: 1.03–2.21 and HR = 1.56, 95% CI: 1.07–2.26, respectively), even after adjusting for confounders. 

Hazard models were used to examine whether the consumption of certain food groups modified the risk of insomnia associated with *CLOCK* rs12649507 in males and females. Table 4(1,2) present whether individuals in the tertile ranges of each food group had exacerbated or mitigated insomnia risk associated with rs12649507. Specifically, the fruit and meat food groups significantly modified the risk of insomnia associated with rs12649507 in males (Table 4(1); *p*_interaction_ = 0.006 in a recessive model for fruit; *p* = 0.010 in a dominant model for meat). Additionally, females with the G allele in the lowest tertile of the beverage intake group had a higher risk of insomnia than those with the AA genotype (Table 4(2); *p* = 0.041 in a dominant model).

Table 5(1,2) present whether food groups modified the insomnia risk associated with *CLOCK* rs4580704 in males and females. Males with the C allele and those in the middle tertile of fruit intake had a significantly reduced risk of insomnia compared to those in the lowest tertile (Table 5(1); HR = 0.64, 95% CI: 0.47–0.86), whereas those with the GG homozygote in the highest tertile of fruit intake had a higher risk of insomnia (Table 5(1); HR = 1.85, 95% CI: 1.05–3.24). Males with the G allele in the lowest tertile of meat intake had reduced insomnia risk, as compared to those with the CC homozygote in the lowest tertile (Table 5(1); HR = 0.51, 95% CI: 0.32–0.80). Additionally, females with CC variants in the middle tertile of beverage intake had a higher risk of insomnia than females in the lowest tertile (Table 5–2; HR = 1.55, 95% CI: 1.04–2.31), and females with the G allele in the lowest tertile of beverage intake had a higher risk of insomnia (Table 5(2); HR = 2.05, 95% CI: 1.39–3.02).

## 4. Discussion

In this longitudinal study, we observed significantly modified insomnia risks depending on the consumption of food groups associated with *CLOCK* rs12649507 and rs4580704 in 775 males and 655 females in the general population. Specifically, males with the *CLOCK* rs12649507 GG homozygote showed a higher risk of developing insomnia than those with the A allele. Furthermore, the fruit and meat food groups modified the risk of insomnia associated with both rs12649507 and rs4580704 in males, whereas in females beverage intake increased the risk of developing insomnia associated with rs12649507 and rs4580704.

Our findings were concurrent with those of previous studies. For example, etiological studies reported that the risk of developing insomnia was connected to the *CLOCK* gene associated with sleep disorders and poor sleep quality due to circadian disruption [14,15,16,17]. A previous study showed that 444 Chinese participants with rs12649507 G homozygotes had poorer sleep quality than those with AA homozygotes [15]. Similarly, the current study consistently found the rs12649507 homozygous G allele to be a minor allele, based on its frequency, that led to a higher risk of insomnia in males. However, another study among a Caucasian population from Central Europe showed a significant link between the *CLOCK* rs12649507 with a minor A allele and shorter sleep duration [14]. Furthermore, climacteric females with an rs12649507 homozygous TT genotype were 1.78 times more likely to have insomnia (95% CI 1.16–2.75) [16].

Additionally, a study showed that rs4580704 was related to the increased energy intake due to the disruption of the circadian rhythm [17]. In this manner, mistimed sleep and wake cycles due to circadian dysregulation resulted in disrupted eating habits [32]. Individuals with the *CLOCK* rs4580704 minor C allele indicated low energy intake [18]. Moreover, meta-analyses studies showed that individuals who obtained less than 5.5 h of sleep per night experienced an upsurge in energy intake compared to those with sufficient sleep [33,34]. Furthermore, decreased sleep changed appetite by increasing cravings for fat-rich foods and snacks [35,36]. In addition, the dysregulation of circadian rhythms may contribute to an increase in cardiometabolic risks. Indeed, *CLOCK* rs4580704 was linked to cardiometabolic risks such as metabolic syndrome [18], obesity [20], diabetes [19], and cardiovascular diseases [19].

Our current findings of the mitigating effects of fruit intake on the insomnia risk associated with the *CLOCK* gene may be attributed to the abundance of antioxidants and vitamins in fruits [37]. A follow-up study among 4476 Mexican teachers indicated that participants in the highest quartile of the fruit and vegetable dietary groups were more likely to have a higher quality of sleep [38]. Another study demonstrated that males who had better sleep quality had higher fruit and vegetable intakes [39].

Regarding the relationship between meat intake and sleep, previous evidence showed not only positive associations with lean meat but also adverse associations with red and processed meat intake. For example, a 9-year longitudinal study among 62,552 Chinese adults aged > 60 years reported that participants who consumed meat daily had better sleep quality due to the favorable effects of an abundance of protein and minerals (including iron and zinc) [37]. In contrast, other studies indicated adverse associations between sleep quality and meat intake; this may have been due mainly to red meat or processed meat products, which have high saturated fat content despite a high favorable protein content. Participants aged > 60 years in the highest tertile of meat intake had a poorer quality of sleep than those in the lowest tertile (OR = 1.71, 95% CI 1.04–2.82) [40].

Additionally, similar to the current study results, a previous study demonstrated that beverage intake showed positive associations with increased sleep disturbances [41]. Future investigations of the risk of insomnia associated with various beverages are needed.

The mechanisms underlying the interaction between food groups and the insomnia risks associated with *CLOCK* rs12649507 and rs4580704 were not completely elucidated in the current study. However, previous studies on the associations between food intake and sleep disorders suggested that favorable effects were attributable to the consumption of fruits rich in antioxidants and vitamins [37], as well as to melatonin synthesized from tryptophan; these dietary components regulated circadian rhythms and aided in the initiation of sleep [38]. As a precursor amino acid, tryptophan synthesizes melatonin via serotonin, which improves sleep quality [34,42].

Our study had both strengths and limitations. First, our study had a large sample size from a general population. Second, the study was a longitudinal follow-up study that took place over 6 years. This enabled us to investigate the contributing and attenuating factors underlying the risk of developing insomnia as well as their interactions. However, this study required additional consideration to interpret our findings. We used the DSM-4, instead of the DSM-5, to define insomnia because the risks of insomnia were compared using consistent criteria from 2005 to 2012.

## 5. Conclusions

To our knowledge, this was the first investigation to longitudinally examine the effect of the consumption of food groups on the associations between *CLOCK* rs12649507 and rs4580704 and the insomnia risk in the large general population. We found that both the fruit and meat food groups significantly mitigated the insomnia risk associated with rs12649507 among males, whereas the beverage group significantly increased the insomnia risk associated with rs12649507 among females. Additionally, among males, the fruit and meat groups significantly modified the insomnia risk associated with rs4580704, while the beverage group exacerbated the risk of insomnia associated with rs4580704 among females. Our findings may help better understand the risk of insomnia to establish practical guidelines based on food consumption related to genetic traits in individuals.

## Figures and Tables

**Table 1:** Baseline characteristics of study participants according to insomnia (*n* = 1430).

	Total (*n* = 1430)			Males (*n* = 775)			Females (*n* = 655)		
	Yes	No		Yes	No		Yes	No	
	*n* = 441 (30.84%)	*n* = 989 (69.16%)	*p*	*n* = 215 (27.74%)	*n* = 560 (72.26%)	*p*	*n* = 226 (34.50%)	*n* = 429 (65.50%)	*p*
Person-years	3.17 ± 1.43	5.48 ± 0.16	<0.001	3.17 ± 1.46	5.49 ± 0.14	<0.001	3.17 ± 1.41	5.47 ± 0.18	<0.001
Age, years	53.11 ± 7.51	51.65 ± 6.69	<0.001	53.30 ± 7.40	51.58 ± 6.49	0.003	52.94 ± 7.63	51.74 ± 6.95	0.043
BMI, kg/m^2^	24.54 ± 2.88	24.65 ± 2.75	0.527	24.66 ± 2.76	24.68 ± 2.55	0.930	24.44 ± 2.99	24.61 ± 2.99	0.493
Smoking, pack-years *	0.00 (0.00, 14.00)	0.00 (0.00, 16.00)	0.046	14.40 (0.00, 27.00)	13.00 (0.00, 26.00)	0.691	0.00 (0.00, 0.00)	0.00 (0.00, 0.00)	0.795
Current drinker, %	47.62	53.79	0.031	72.56	73.39	0.814	23.89	28.21	0.236
Physical activity, MET *	11.40 (0.00, 32.00)	14.00 (0.00, 32.00)	0.378	14.00 (0.00, 32.00)	16.00 (0.00, 34.60)	0.716	11.40 (0.00, 27.50)	11.40 (0.00, 30.00)	0.541
Depression index, BDI *	6.00 (3.00, 11.00)	4.00 (2.00, 8.00)	<0.001	5.00 (3.00, 11.00)	4.00 (1.00, 7.00)	<0.001	6.00 (4.00, 11.00)	5.00 (3.00, 9.00)	<0.001
Total caloric intake	1869.00 ± 528.96	1912.55 ± 512.07	0.142	2021.22 ± 541.37	2034.12 ± 508.93	0.756	1724.20 ± 474.56	1753.85 ± 471.62	0.446
C-RP *	0.65 (0.34, 1.40)	0.59 (0.33, 1.32)	0.305	0.71 (0.37, 1.43)	0.68 (0.37, 1.43)	0.518	0.58 (0.31, 1.38)	0.51 (0.28, 1.19)	0.166
Hypertension, %	41.04	36.91	0.137	44.65	40.54	0.298	37.61	32.17	0.162
Diabetes mellitus, %	17.91	14.76	0.131	20.00	18.04	0.529	15.93	10.49	0.044

Mean ± SD; BMI, body mass index; C-RP, C-reactive protein; BDI, Beck Depression Index; * median (interquartile range) Kruskal-Wallis Test.

**Table 2:** Genetic information of the CLOCK gene polymorphisms in this study.

Gene	Location in Chromosome	rs Number	Major/Minor Allele	Position	MAF	HWE *p*-Value
*CLOCK*	Intron 4	rs12649507	A > G	56,075,241	0.3598	0.3556
	Intron 4	rs4580704	C > G	56,021,464	0.2589	0.5794

MAF, minor allele frequency; HWE, Hardy–Weinberg equilibrium.

**Table 3:** Associations between the *CLOCK* gene and insomnia risk.

		Hazard Ratio (95% Confidence Intervals)		
		Total (*n* = 1430)	Males (*n* = 775)	Females (*n* = 655)
rs12649507				
Additive	Wild, AA	1.00 (ref)	1.00 (ref)	1.00 (ref)
	Hetero, GA	1.03 (0.86, 1.22)	0.81 (0.63, 1.04)	1.26 (0.99, 1.61)
	Homo, GG	1.21 (0.95, 1.54)	1.35 (0.98, 1.86)	1.05 (0.72, 1.51)
Dominant	GA + GG	1.06 (0.90, 1.25)	0.93 (0.74, 1.17)	1.22 (0.97, 1.54)
	AA	1.00 (ref)	1.00 (ref)	1.00 (ref)
Recessive	GG	1.19 (0.95, 1.49)	1.50 (1.11, 2.02)	0.91 (0.65, 1.28)
	AA + GA	1.00 (ref)	1.00 (ref)	1.00 (ref)
rs4580704				
Additive	Wild, CC	1.00 (ref)	1.00 (ref)	1.00 (ref)
	Hetero, GC	1.10 (0.93, 1.29)	0.92 (0.72, 1.18)	1.24 (0.99, 1.56)
	Homo, GG	1.27 (0.94, 1.72)	1.51 (1.03, 2.21)	0.98 (0.59, 1.61)
Dominant	GC + GG	1.12 (0.96, 1.31)	1.01 (0.81, 1.27)	1.21 (0.97, 1.51)
	CC	1.00 (ref)	1.00 (ref)	1.00 (ref)
Recessive	GG	1.22 (0.91, 1.64)	1.56 (1.07, 2.26)	0.88 (0.54, 1.44)
	CC + GC	1.00 (ref)	1.00 (ref)	1.00 (ref)

Adjusted for age, energy, body mass index, sex, smoking, drinking, physical activity, depression, C-reactive protein, hypertension, and diabetes.

**Table 4:** (**1**) Interactions according to tertiles of food group consumption with the associations between *CLOCK* rs12649507 and insomnia among males (*n* = 775). (**2**) among females (*n* = 655).

(**1**)							
**Food Groups**		**rs12649507**					
		**Dominant**			**Recessive**		
		**GA + GG**	**AA**	***p***	**GG**	**GA + AA**	***p***
Refined grains	T1	1.13 (0.73, 1.74)	1.00 (ref)	0.280	1.09 (0.62, 1.94)	1.00 (ref)	0.936
	T2	0.92 (0.58, 1.44)	1.03 (0.64, 1.65)		2.10 (1.27, 3.49)	0.78 (0.56, 1.08)	
	T3	1.22 (0.80, 1.88)	1.46 (0.94, 2.26)		1.48 (0.90, 2.45)	1.21 (0.90, 1.63)	
Mixed grains	T1	0.79 (0.54, 1.15)	1.00 (ref)	0.654	1.29 (0.79, 2.09)	1.00 (ref)	0.954
	T2	0.76 (0.51, 1.11)	0.64 (0.42, 0.99)		1.63 (0.99, 2.69)	0.74 (0.54, 1.02)	
	T3	0.75 (0.50, 1.11)	0.85 (0.55, 1.31)		1.10 (0.62, 1.95)	0.92 (0.67, 1.27)	
Vegetables	T1	0.91 (0.62, 1.35)	1.00 (ref)	0.326	1.39 (0.84, 2.29)	1.00 (ref)	0.700
	T2	0.75 (0.50, 1.13)	1.02 (0.68, 1.52)		1.41 (0.84, 2.39)	0.91 (0.68, 1.23)	
	T3	0.80 (0.54, 1.19)	0.63 (0.39, 1.01)		1.21 (0.71, 2.04)	0.75 (0.55, 1.04)	
Fruits	T1	0.80 (0.55, 1.16)	1.00 (ref)	0.963	0.69 (0.38, 1.23)	1.00 (ref)	0.006
	T2	0.71 (0.47, 1.06)	0.56 (0.36, 0.86)		1.61 (0.95, 2.73)	0.61 (0.44, 0.83)	
	T3	0.72 (0.49, 1.07)	0.94 (0.62, 1.43)		1.53 (0.96, 2.43)	0.78 (0.58, 1.06)	
Meats	T1	0.54 (0.36, 0.81)	1.00 (ref)	0.010	1.22 (0.66, 2.24)	1.00 (ref)	0.718
	T2	0.83 (0.58, 1.18)	0.63 (0.42, 0.96)		1.65 (1.02, 2.67)	0.92 (0.67, 1.26)	
	T3	0.83 (0.59, 1.18)	0.73 (0.47, 1.13)		1.49 (0.91, 2.43)	1.02 (0.75, 1.39)	
Seafood	T1	0.71 (0.48, 1.04)	1.00 (ref)	0.259	1.50 (0.93, 2.43)	1.00 (ref)	0.556
	T2	0.71 (0.49, 1.04)	0.58 (0.38, 0.90)		1.42 (0.86, 2.33)	0.77 (0.56, 1.06)	
	T3	0.79 (0.54, 1.16)	0.83 (0.55, 1.25)		1.23 (0.68, 2.21)	1.03 (0.76, 1.39)	
Milk and dairy	T1	0.85 (0.58, 1.26)	1.00 (ref)	0.238	1.44 (0.82, 2.55)	1.00 (ref)	0.889
	T2	1.17 (0.81, 1.68)	0.74 (0.47, 1.18)		1.72 (1.08, 2.76)	1.05 (0.77, 1.43)	
	T3	0.67 (0.45, 1.01)	1.12 (0.75, 1.68)		1.27 (0.75, 2.16)	0.91 (0.67, 1.25)	
Beverages	T1	1.14 (0.74, 1.76)	1.00 (ref)	0.163	1.30 (0.75, 2.24)	1.00 (ref)	0.997
	T2	1.11 (0.71, 1.74)	1.17 (0.74, 1.86)		2.08 (1.20, 3.60)	1.00 (0.73, 1.38)	
	T3	1.22 (0.79, 1.90)	1.61 (1.01, 2.55)		1.67 (1.02, 2.75)	1.25 (0.90, 1.73)	
(**2**)							
**Food Groups**		**rs12649507**					
		**Dominant**			**Recessive**		
		**GA + GG**	**AA**	***p***	**GG**	**GA + AA**	***p***
Refined grains	T1	1.14 (0.79, 1.63)	1.00 (ref)	0.570	0.83 (0.49, 1.40)	1.00 (ref)	0.414
	T2	0.98 (0.67, 1.43)	0.79 (0.51, 1.23)		0.63 (0.32, 1.26)	0.83 (0.63, 1.10)	
	T3	0.86 (0.59, 1.26)	0.64 (0.40, 1.04)		0.82 (0.46, 1.47)	0.69 (0.52, 0.92)	
Mixed grains	T1	1.27 (0.85, 1.90)	1.00 (ref)	0.251	0.65 (0.32, 1.34)	1.00 (ref)	0.825
	T2	1.49 (1.01, 2.21)	0.97 (0.59, 1.57)		1.34 (0.81, 2.21)	1.04 (0.78, 1.39)	
	T3	0.97 (0.64, 1.48)	1.07 (0.68, 1.68)		0.67 (0.35, 1.26)	0.86 (0.64, 1.16)	
Vegetables	T1	1.67 (1.06, 2.62)	1.00 (ref)	0.068	0.74 (0.36, 1.53)	1.00 (ref)	0.109
	T2	1.68 (1.07, 2.62)	1.36 (0.81, 2.30)		0.75 (0.41, 1.38)	1.15 (0.86, 1.54)	
	T3	1.60 (1.01, 2.53)	1.65 (1.03, 2.66)		1.49 (0.89, 2.49)	1.07 (0.80, 1.43)	
Fruits	T1	1.03 (0.68, 1.54)	1.00 (ref)	0.326	0.97 (0.56, 1.68)	1.00 (ref)	0.694
	T2	1.03 (0.68, 1.55)	0.82 (0.51, 1.30)		0.57 (0.26, 1.23)	0.97 (0.72, 1.29)	
	T3	1.20 (0.80, 1.79)	0.88 (0.55, 1.39)		1.17 (0.68, 1.99)	1.03 (0.77, 1.38)	
Meats	T1	1.38 (0.90, 2.12)	1.00 (ref)	0.189	0.71 (0.35, 1.48)	1.00 (ref)	0.842
	T2	1.49 (0.98, 2.28)	1.07 (0.65, 1.75)		1.21 (0.69, 2.13)	1.02 (0.76, 1.37)	
	T3	1.45 (0.95, 2.21)	1.51 (0.95, 2.39)		1.00 (0.58 1.70)	1.18 (0.89, 1.58)	
Seafood	T1	1.82 (1.16, 2.84)	1.00 (ref)	0.105	0.98 (0.55, 1.77)	1.00 (ref)	0.491
	T2	1.54 (0.98, 2.44)	1.62 (0.99, 2.66)		0.49 (0.20, 1.20)	1.10 (0.83, 1.46)	
	T3	1.51 (0.96, 2.39)	1.40 (0.86, 2.29)		1.14 (0.70, 1.86)	0.94 (0.70, 1.25)	
Milk and dairy	T1	1.47 (0.97, 2.22)	1.00 (ref)	0.185	0.91 (0.53, 1.55)	1.00 (ref)	0.775
	T2	1.40 (0.92, 2.15)	1.15 (0.71, 1.85)		0.79 (0.38, 1.65)	1.01 (0.76, 1.35)	
	T3	1.27 (0.82, 1.95)	1.27 (0.79, 2.03)		0.99 (0.56, 1.75)	0.96 (0.72, 1.30)	
Beverages	T1	1.63 (1.08, 2.47)	1.00 (ref)	0.041	0.97 (0.59, 1.59)	1.00 (ref)	0.642
	T2	1.52 (0.99, 2.33)	1.24 (0.77, 1.99)		1.00 (0.50, 2.00)	1.00 (0.75, 1.33)	
	T3	1.16 (0.75, 1.81)	1.32 (0.81, 2.13)		0.70 (0.36, 1.34)	0.89 (0.67, 1.20)	

Adjusted for age, energy, body mass index, sex, smoking, drinking, physical activity, depression, C-reactive protein, hypertension, and diabetes; *p*, *pinteraction*.

**Table 5:** (**1**) Interactions according to tertiles of food group consumption on the associations between *CLOCK* rs4580704 and insomnia among males (*n* = 775). (**2**) among females (*n* = 655).

(**1**)							
**Food Groups**		**rs4580704**					
		**Dominant**			**Recessive**		
		**CG + GG**	**CC**	***p***	**GG**	**CC + CG**	***p***
Refined grains	T1	1.07 (0.71, 1.62)	1.00 (ref)	0.982	0.92 (0.44, 1.91)	1.00 (ref)	0.263
	T2	0.85 (0.54, 1.34)	0.97 (0.65, 1.44)		2.25 (1.12, 4.52)	0.82 (0.60, 1.13)	
	T3	1.32 (0.89, 1.95)	1.23 (0.84, 1.79)		1.92 (1.08, 3.41)	1.16 (0.87, 1.55)	
Mixed grains	T1	1.08 (0.74, 1.56)	1.00 (ref)	0.415	2.03 (1.17, 3.53)	1.00 (ref)	0.160
	T2	0.88 (0.59, 1.33)	0.80 (0.55, 1.17)		1.29 (0.62, 2.68)	0.84 (0.62, 1.14)	
	T3	0.85 (0.55, 1.30)	1.00 (0.68, 1.46)		1.02 (0.49, 2.12)	0.96 (0.71, 1.31)	
Vegetables	T1	1.05 (0.71, 1.53)	1.00 (ref)	0.507	1.70 (0.95, 3.05)	1.00 (ref)	0.938
	T2	0.81 (0.54, 1.23)	1.03 (0.72, 1.46)		1.21 (0.60, 2.42)	0.95 (0.71, 1.27)	
	T3	0.91 (0.61, 1.34)	0.70 (0.47, 1.03)		1.31 (0.66, 2.61)	0.78 (0.58, 1.06)	
Fruits	T1	0.78 (0.54, 1.13)	1.00 (ref)	0.138	0.57 (0.25, 1.29)	1.00 (ref)	0.006
	T2	0.71 (0.47, 1.07)	0.61 (0.42, 0.89)		1.77 (0.94, 3.31)	0.64 (0.47, 0.86)	
	T3	0.89 (0.61, 1.30)	0.77 (0.53, 1.11)		1.85 (1.05, 3.24)	0.82 (0.61, 1.09)	
Meats	T1	0.51 (0.32, 0.80)	1.00 (ref)	0.001	0.98 (0.43, 2.25)	1.00 (ref)	0.373
	T2	0.93 (0.64, 1.35)	0.71 (0.50, 1.02)		1.90 (1.01, 3.58)	0.93 (0.69, 1.26)	
	T3	1.00 (0.70, 1.41)	0.71 (0.48, 1.04)		1.66 (0.94, 2.92)	1.01 (0.75, 1.36)	
Seafood	T1	0.85 (0.57, 1.25)	1.00 (ref)	0.199	1.13 (0.57, 2.24)	1.00 (ref)	0.375
	T2	0.75 (0.50, 1.12)	0.74 (0.51, 1.07)		1.47 (0.78, 2.75)	0.76 (0.56, 1.02)	
	T3	1.01 (0.69, 1.47)	0.84 (0.58, 1.22)		1.62 (0.87, 3.04)	0.94 (0.71, 1.26)	
Milk and dairy	T1	0.80 (0.53, 1.22)	1.00 (ref)	0.733	1.29 (0.60, 2.81)	1.00 (ref)	0.642
	T2	1.31 (0.92, 1.87)	0.78 (0.53, 1.14)		1.68 (0.97, 2.91)	1.05 (0.78, 1.41)	
	T3	0.71 (0.48, 1.07)	0.98 (0.68, 1.40)		1.51 (0.76, 3.02)	0.90 (0.67, 1.21)	
Beverages	T1	1.17 (0.77, 1.77)	1.00 (ref)	0.474	1.28 (0.64, 2.55)	1.00 (ref)	0.974
	T2	1.09 (0.70, 1.70)	1.15 (0.78, 1.70)		2.32 (1.26, 4.30)	0.99 (0.73, 1.36)	
	T3	1.31 (0.86, 1.99)	1.39 (0.93, 2.06)		1.61 (0.83, 3.12)	1.25 (0.92, 1.71)	
(**2**)							
**Food Groups**		**rs4580704**					
		**Dominant**			**Recessive**		
		**CG + GG**	**CC**	***p***	**GG**	**CC + CG**	***p***
Refined grains	T1	1.07 (0.76, 1.51)	1.00 (ref)	0.133	0.79 (0.32, 1.95)	1.00 (ref)	0.412
	T2	0.90 (0.63, 1.30)	0.82 (0.56, 1.18)		0.54 (0.20, 1.48)	0.84 (0.64, 1.10)	
	T3	0.94 (0.66, 1.33)	0.57 (0.38, 0.85)		0.89 (0.43, 1.85)	0.70 (0.53, 0.93)	
Mixed grains	T1	1.23 (0.84, 1.81)	1.00 (ref)	0.451	0.63 (0.23, 1.71)	1.00 (ref)	0.427
	T2	1.45 (1.00, 2.10)	1.04 (0.70, 1.56)		1.04 (0.45, 2.38)	1.11 (0.84, 1.46)	
	T3	0.96 (0.63, 1.45)	0.97 (0.66, 1.43)		0.92 (0.41, 2.04)	0.85 (0.64, 1.14)	
Vegetables	T1	1.63 (1.08, 2.46)	1.00 (ref)	0.114	0.00 (-, -)	1.00 (ref)	-
	T2	1.55 (1.02, 2.37)	1.38 (0.90, 2.10)		0.43 (0.16, 1.18)	1.14 (0.86, 1.51)	
	T3	1.52 (1.00, 2.33)	1.48 (0.99, 2.23)		2.60 (1.44, 4.69)	1.03 (0.78, 1.37)	
Fruits	T1	1.13 (0.77, 1.65)	1.00 (ref)	0.902	0.70 (0.28, 1.75)	1.00 (ref)	0.237
	T2	1.18 (0.80, 1.74)	0.83 (0.55, 1.24)		0.40 (0.10, 1.64)	0.93 (0.71, 1.23)	
	T3	1.18 (0.80, 1.75)	1.07 (0.73, 1.57)		1.31 (0.68, 2.54)	1.01 (0.76, 1.33)	
Meats	T1	1.17 (0.78, 1.74)	1.00 (ref)	0.547	0.41 (0.10, 1.68)	1.00 (ref)	0.133
	T2	1.43 (0.97, 2.10)	0.94 (0.63, 1.41)		0.69 (0.22, 2.18)	1.06 (0.80, 1.41)	
	T3	1.29 (0.88, 1.91)	1.28 (0.87, 1.88)		1.36 (0.74, 2.51)	1.14 (0.86, 1.51)	
Seafood	T1	1.46 (0.99, 2.14)	1.00 (ref)	0.529	0.90 (0.33, 2.48)	1.00 (ref)	0.228
	T2	1.24 (0.84, 1.85)	1.26 (0.86, 1.85)		0.18 (0.03, 1.29)	1.09 (0.83, 1.43)	
	T3	1.29 (0.88, 1.89)	1.06 (0.71, 1.57)		1.30 (0.71, 2.39)	0.94 (0.71, 1.24)	
Milk and dairy	T1	1.18 (0.81, 1.72)	1.00 (ref)	0.844	1.05 (0.51, 2.16)	1.00 (ref)	0.813
	T2	1.19 (0.82, 1.72)	0.99 (0.67, 1.45)		0.50 (0.12, 2.06)	1.03 (0.78, 1.36)	
	T3	1.17 (0.81, 1.71)	0.94 (0.64, 1.39)		0.92 (0.42, 2.02)	0.99 (0.74, 1.32)	
Beverages	T1	2.05 (1.39, 3.02)	1.00 (ref)	0.004	1.01 (0.54, 1.89)	1.00 (ref)	0.763
	T2	1.45 (0.95, 2.22)	1.55 (1.04, 2.31)		0.37 (0.05, 2.64)	1.02 (0.78, 1.34)	
	T3	1.24 (0.81, 1.91)	1.37 (0.90, 2.07)		0.79 (0.32, 1.96)	0.88 (0.66, 1.17)	

Adjusted for age, energy, body mass index, sex, smoking, drinking, physical activity, depression, C-reactive protein, hypertension, and diabetes; *p*, *p_interaction_*.

## Supplementary Materials

The following are available online at https://www.mdpi.com/article/10.3390/nu15102300/s1, Table S1: Food groups and the included food items.
