# Supplementary material for: Association between CLOCK Gene Polymorphisms and Insomnia Risk According to Food Groups: A KoGES Longitudinal Study
Source: Nutrients. 2023 May 13;15(10):2300. doi: 10.3390/nu15102300 (PMC10222773; doi:10.3390/nu15102300)
Supplement: Supplementary file 1 [file nutrients-15-02300-s001.zip › nutrients-2282720-supplementary.pdf]

Supplemental Table S1. Food groups and the included food items

Food groups	Included food items
Refined grains	rice, ramen, noodles, dumpling, rice cake, bread, jam, cake, cookie
Mixed grains	whole grain rice with beans, multi-grains, rice roll
Vegetables	cabbage, spinach, zucchini, seasoned bracken, cucumber, radish, salad, green onion, lettuce, broccoli, tomato
Fruits	strawberry, melon, peach, grape, apple, pear, persimmon, tangerine, banana, orange, kiwi
Meats	pork, beef, chicken, egg
Seafood	mackerel, grilled yellow croaker, anchovy, squid, crab, shrimp
Milk and dairy	milk, yogurt, ice cream, cheese
Beverages	soy milk, coffee, soft drink, green tea, other beverages
